# Extension of Poultry Meat Shelf Life Using *Cynara cardunculus* L. Leaf Extracts as a Natural Preservative

**DOI:** 10.3390/foods14152592

**Published:** 2025-07-24

**Authors:** Cássia H. Barbosa, Mariana A. Andrade, Fernanda Vilarinho, Ana Sanches Silva, Ana Luísa Fernando

**Affiliations:** 1 Department of Food and Nutrition, National Institute of Health Doutor Ricardo Jorge, Av. Padre Cruz, 1649-016 Lisbon, Portugal; cassia.barbosa@insa.min-saude.pt (C.H.B.); mariana.andrade@insa.min-saude.pt (M.A.A.); fernanda.vilarinho@insa.min-saude.pt (F.V.); 2 MEtRICs, Department of Chemistry, NOVA School of Science and Technology, Universidade NOVA de Lisboa, Campus de Caparica, 2829-516 Caparica, Portugal; 3 REQUIMTE/LAQV, Rua D. Manuel II, Apartado 55142, Porto, Portugal; 4 Faculty of Pharmacy, University of Coimbra, Azinhaga de Santa Comba, 3000-548 Coimbra, Portugal; 5 Centre for Study in Animal Science (CECA), ICETA, University of Porto, 4501-401 Porto, Portugal; 6 Associate Laboratory for Animal and Veterinary Sciences (AL4AnimalS), 1300-477 Lisbon, Portugal

**Keywords:** cultivated cardoon, globe artichoke, natural additive, antimicrobial, antioxidant, lipid oxidation

## Abstract

Food additives are used to prevent food spoilage and extend its shelf life. However, concerns regarding the potential health implications associated with some synthetic additives have prompted research efforts aimed at identifying natural alternatives, such as plant extracts. *Cynara cardunculus* L. (cardoon) is known for its antimicrobial and antioxidant properties. The aim of this study was to evaluate the capability of ethanolic food-grade extracts from cultivated cardoon and globe artichoke leaves to preserve poultry breast meat during refrigerated storage. A total of seven treatment groups were tested: one control group (no extract) and six active groups with 0.5%, 1%, and 2% (*w*/*w*) of either cultivated cardoon or globe artichoke leaf extracts. Lipid oxidation, moisture, colour, pH, acidity, and microbial growth were assessed in poultry meat samples over 15 days. Both extracts were effective in extending shelf life, up to 11 days, by delaying lipid oxidation and microbial growth. Cardoon extract (1% *w*/*w*) displayed superior antimicrobial efficacy, maintaining microbial counts below 5 Log CFU/g meat until day 15, compared to the control. Cultivated cardoon leaf extract proves promising as a natural antimicrobial and antioxidant, extending the shelf life of poultry meat. This presents an opportunity to maintain the quality of meat products, aligning with consumer preferences for natural ingredients and sustainable practices.

## 1. Introduction

Poultry meat is the most consumed meat in the world [1] and is valued for its low cost and nutritional value. Despite that, poultry meat is a very perishable food, prone to lipid oxidation and microbial degradation, which affects its sensory, nutritional, and safety characteristics and reduces its shelf life. To mitigate this issue, the food industry has been looking for ways to preserve food and extend its shelf life. Food additives can help extend foods’ shelf life by delaying foods’ natural deterioration. In the European Union, according to Regulation (EC) No 1333/2008 [2], food additives are “any substance not normally consumed as a food in itself and not normally used as a characteristic ingredient of food, whether or not it has nutritive value, the intentional addition of which to food for a technological purpose in the manufacture, processing, preparation, treatment, packaging, transport or storage of such food results, or may be reasonably expected to result, in it or its by-products becoming directly or indirectly a component of such foods”. A food additive can have a natural (from animals or plants) or a synthetic origin [3]. The application of synthetic additives has grown with the advance of technologies, and despite the strict European Union regulations on the approval and application of food additives, some have been associated with several diseases/disorders, and their long-term effects are still unknown [4,5,6,7,8,9]. For instance, synthetic antioxidant additives, such as the most commonly used, butylated hydroxytoluene (BHT) and butylated hydroxyanisole (BHA), have been associated with toxicity, cancer, and metabolic disorders. Also, commonly used synthetic antimicrobials, like nitrates, nitrites, and sulphites, have been identified as promoters of cancer and toxicity [4,5,9,10,11,12]. The usage of natural additives has been explored, in order to avoid the adverse effects of synthetic additives and increase population awareness concerning food additives and their associated risks. Ascorbic acid, tocopherols, natamycin, nisin, and carotenoids are some of the natural additives that are currently approved for use [3,5,9,12]. Nonetheless, researchers are currently studying other sources of natural compounds, which could potentially be incorporated into food products as additives, namely essential oils and extracts from plants and agro-industrial by-products, generally recognized as excellent sources of antioxidants and antimicrobials. Table 1 presents some examples of natural extracts used in poultry meat and poultry meat products. For example, plum (*Prunus salicina*) pulp and peel microparticles were added to breast chicken patties [13] and were able to reduce the lipid oxidation up to 50% by the end of 10-day storage at 4 °C, as well as increase the antioxidant capacity and levels of α- and γ-tocopherols in the samples [13].

*Cynara cardunculus* L., also recognised as cardoon, is native to the Mediterranean region and is classified as part of the Asteraceae family. It consists of three varieties: wild cardoon (var. *sylvestris* (Lamk) Fiori), cultivated cardoon (var. *altilis*), and globe artichoke (var. *scolymus* (L.) Fiori). Cardoon is a valuable industrial crop since it can develop in environmentally challenging conditions, like soil poor in nutrients and low water availability, and still have a high production yield [4,14,15,16]. In addition, it is a multipurpose crop with applications in several areas. The edible parts (immature heads and fleshy stems) are used in Mediterranean cuisine in the preparation of soups and salads [4]. Its flowers are used as a vegetable coagulant to produce various kinds of cheeses in different countries such as Portugal, Italy, and Spain. Cardoon by-products, particularly the leaves, are rich in bioactive compounds like silymarin and cynarine, and have been applied in traditional medicine. In addition, due to their bioactive compounds content, cardoon leaves present promising antioxidant and antimicrobial properties [4,14,17]. Therefore, the cardoon leaves generated by the cheese-making process can be applied as natural food additives, helping to reduce the use of synthetic additives in foods. In addition, using a by-product from the cardoon crop helps to achieve the targets of the United Nations Sustainable Development Goals, namely Goal 12 (Responsible Consumption and Reduction), which emphasises reducing waste production through prevention, reduction, recycling, and reuse [18]. The direct addition of natural additives derived from these by-products can increase the shelf life of food, thus reducing food loss and promoting the consumption of food with fewer synthetic additives. The development of such additives considers sustainable practices, including the valorisation of agricultural by-products and the reduction of food waste. For example, Mazzaglia et al. [19] successfully used *Cynara cardunculus* extract as an additive in aubergine-based burgers to extend their shelf life, and Mirpoor et al. [16] used cardoon leaf extract to develop an active film with good mechanical and barrier properties and noteworthy antioxidant activity. In addition, Mirpoor et al. [20] were able to protect peanuts from deterioration with a protein-based film from cardoon proteins extracted from cardoon oilseed waste. Despite their potential bioactivity, limited research has investigated and compared the effects of cardoon and artichoke leaf extracts on the quality and shelf life of food products, leaving a gap in their application as natural preservatives.

The direct application of natural food additives is a strategy to prolong the foodsʹ product shelf life due to their antioxidant and antimicrobial potential. Adding the natural additive directly to the food has the advantage of being more effective in limiting its oxidation, as well as protecting it from stress factors such as light and temperature [21,22]. This study aims to compare the application of two different extracts (cultivated cardoon leaves and globe artichoke leaves) in maintaining the quality of poultry breast meat over a 15-day refrigerated storage period at 5 ± 2 °C, in comparison with untreated poultry breast meat samples. Considering its high polyunsaturated fatty acid (PUFA) content, poultry meat was selected as a model food to assess the efficacy of cultivated cardoon leaf extract and globe artichoke leaf extract on inhibiting its deterioration. These extracts were chosen as they present potential antioxidant and antimicrobial activity, as described in our previous study [23]. The cardoon extract presented a total phenolic content (TPC) of 82 mg GAE/g extract, a total flavonoid content (TFC) of 145 mg ECE/g extract, and the strongest antioxidant capacity (EC_50_ = 2.1 mg/mL). In contrast, the globe artichoke extract showed lower values: a TPC of 49 mg GAE/g, a TFC of 81 mg ECE/g, and an EC_50_ of 3.9 mg/mL. Phenolic profiling by UHPLC-ToF-MS confirmed the presence of chlorogenic acid, luteolin, and apigenin as the major compounds in both extracts [23].

**Table 1:** Studies using natural extracts to preserve poultry meat products.

Natural Extract	Concentration	Poultry Product	Experiment Conditions	Main Results	Reference
Plum (*Prunus salicina*) peel and pulp microparticles extracts	2.0%	Raw breast chicken patties	The extracts were mixed separately with the poultry meat, placed on expanded polystyrene trails, covered by polyethylene films, and stored, in the dark, at 4 °C for 10 days.	The extracts were effective as an antioxidant additive as the antioxidant capacity and tocopherol levels were enhanced. In addition, the lipid oxidation was delayed, and colour and texture features were improved.	[13]
Pomegranate peel (*Punica granatum* L.) extract (PPE) and olive leaf (*Olea europaea* L.) extract (OLE)	10 mg of PPE and 0.25 mg of OLE for a gram of meat	Minced poultry meat	PPE and OLE were blended with the poultry meat and refrigerated (4 °C) over a storage period of 6 days.	The extracts reduced the colony count during the 6-day storage, preserved the lipid oxidation, and maintained the pH levels.	[24]
Olive leaf extract OLE	0.25%, 0.5%, and 1%	Poultry meat slices from chicken breast	Samples were dipped in OLE aqueous solution at the different concentrations and stored at 4 ± 1 °C for 15 days.	The extracts delayed microbial growth compared to the control and maintained chemical quality, with stable pH levels and lower TVN and TBA values. The samples with 1% OLE presented the best results.	[25]
Green tea (*Camellia sinensis*) extract GTE	0.5% and 1%	Chicken meat patties	Samples were mixed with the extract at different concentrations, packaged in high-density polyethylene bags, and stored at 4 °C for 10 days.	The addition of the extracts to the patties delayed lipid oxidation, but did not delay the microbial growth. The suggested concentration of GTE is 0.5% as it improved colour stability and did not impact the sensory attributes.	[26]
*Moringa oleifera* leaf extract (MOLE)	0.25%, 0.5%, 0.7%, and 1%	Chicken breasts	Samples were mixed with the extract at different concentrations and wrapped with polyethylene plastic for 6 days and stored at 4 ± 1 °C and 25 °C.	MOLE was effective in reducing microbial growth (aerobic count, *E. coli, L. monocytogenes*, and *Salmonella* spp.). MOLE at 0.25% was more effective against *E. coli* and *L. monocytogenes.* For aerobic count, both 0.25% and 0.5% were effective, maintaining the levels below spoilage levels.	[27]
Grape extract Green tea extract Rosemary extract Pomegranate extract Mate extract	0.125%, 0.25%, 0.5%, and 1%	Mechanically deboned poultry meat	The extracts were mixed with poultry meat, packed in transparent polyethylene bags, and stored at 2 °C for 10 days.	All extracts were able to reduce the TBA values, with 1% grape extract presenting the best result. All extracts were able to decrease the pH values, with the decrease being more pronounced with increasing extract concentration.	[28]
*Cymbopogon citratus* extract	0.5% and 1%	Chicken sausage	The extracts were mixed with poultry meat, soaked in natural pork casings, packed in polystyrene trays, coated with polyvinyl chloride film paper, and stored at 4 °C for 42 days.	The extract decreased the pH of chicken sausage during storage but did not influence the a_W_ values. The extract maintained low TBA values and preserved the colour of the samples throughout the assay. The extract did not affect the sausage; it demonstrated good acceptability by the consumer.	[29]
Coriander (*Coriandrum sativum* L.) extract	1%	Poultry meat patties	The extracts were mixed with poultry meat and stored under refrigeration (4 °C) for 9 days.	The coriander extract improved the total phenolic content of the patties and was able to maintain significantly lower TBA and peroxide values, as well as carbonyl values. The total plate count was significantly lower for the patties with coriander extract and below acceptable limits.	[30]
Allspice leaf essential oil	500 mg/mL and 1000 mg/mL	Mechanically deboned poultry meat (MDPM)	The MDPM was blended with different concentrations of allspice essential oil, wrapped in plastic bags without vacuum, and kept at 2 °C for 10 days.	Allspice leaf essential oil in both concentrations presented similar results, with low TBA values compared to the control sample. The essential oil was also able to control the decrease of the pH levels during storage, compared to the control.	[31]
Black mulberry leaf extract (BMLE)	0.1%, 0.3%, and 0.5%	Raw chicken meat	The chicken meat was soaked in distilled water with varying levels of BMLE for 5 min, drained for 5 min, then placed in polystyrene trays and covered with polyethylene film, and kept at 4 °C for 12 days.	BMLE was effective in prolonging chicken meat shelf life as it delayed microbial growth and lipid oxidation. Also, the extract reduced the production of biogenic amine and helped maintain the of the meat compared to the control.	[32]
Moringa and olive leaves and extracts	1% and 2% for powder and 0.02% for extracts	Chicken burgers	The chicken meat was minced and mixed with the active compound, wrapped in polyethylene plastic, and stored in a refrigerator at 4 ± 1 °C, for 20 days.	Moringa leaf was able to delay lipid oxidation of chicken burgers and formation of TVN. In addition, both extracts did not negatively affect sensory properties (colour, flavour, odour, texture, or overall acceptability).	[33]
Peppermint essential oil	0.5 and 1%	Broiler chicken meat	The chicken meat was sprayed with the essential oil, vacuum-packed, subjected to ultrasound for 2, 4, and 6 min, and stored for 12 days at 4 °C.	The extract, in combination with ultrasonication, proved to be capable of delaying lipid oxidation, microbial growth, TVN, and pH values. The combination of 1% peppermint essential oil with a 6 min ultrasonication presented the best result.	[34]

BMLE—black mulberry leaf extract; GTE—green tea extract; MDPM—mechanically deboned poultry meat; MOLE—*Moringa oleifera* leaf extracts; OLE—olive leaf extract; TBA—thiobarbituric acid; TVN—total volatile nitrogen.

## 2. Materials and Methods

### 2.1. Reagents

Absolute ethanol ACS reagent for analysis was sourced from Merck (Darmstadt, Germany), 1,1,3,3-tetraethoxypropane (TEP) was obtained from Sigma-Aldrich (Steinheim, Germany), and sodium hydroxide (NaOH) was supplied by Alfa Aesar (Kandel, Germany). Reagents including sodium chloride (NaCl), 2-thiobarbituric acid (TBA), phenolphthalein, boric acid, hydrochloric acid, and trichloroacetic acid (TCA) were purchased from PanReac (Barcelona, Spain). Violet red bile glucose (VRBG), tryptone, and plate count agar (PCA) were provided by Biokar (Allonne, Beauvais, France). All chemicals are of analytical reagent grade. Purified water using the Milli-Q system (Millipore, Belford, MA, USA) was used.

### 2.2. Sample Preparation

*Cynara cardunculus* var. *scolymus* (globe artichoke) dry leaves were acquired at a local shop, and upon arrival at the laboratory, the leaves were ground, vacuum packaged, and stored at room temperature (±21 °C) and protected from light until use. The Portuguese company NINA, Lda, (Guarda, Portugal) kindly supplied the *Cynara cardunculus* var. *altilis* (cultivated cardoon) leaves. The cultivated cardoon leaves were dried out in an oven with controlled air flow at 35 °C, and then ground, vacuum packaged, protected from light, and kept at room temperature until use.

A solid–liquid extraction was carried out following the method described by Andrade et al. [35]. Ethanol was used as the solvent, since it is a food-grade solvent [36]. Briefly, in a centrifuge tube, the sample and ethanol were mixed in a proportion of 1:10 (*w*/*v*), homogenised for 30 min, and then centrifuged (Eppendorf AG 5804R centrifuge, Hamburg, Germany) for 10 min at 11,952× *g*, 10 °C. The supernatant was moved to an evaporation amber pear-shaped flask for evaporation at 35 °C until dryness, in a rotary evaporator (Büchi model R-210, Labortechnik, Flawil, Switzerland). The obtained extract was vacuum-packed and stored at −20 °C until further use.

### 2.3. Application of Extracts in Fresh Poultry Meat

Fresh poultry breast meat was acquired from a nearby market in Lisbon, then ground using a domestic kitchen mixer. After homogenisation, the meat was divided into seven groups: the control group (without extract) and six treatment groups containing cardoon or globe artichoke leaf extracts at concentrations of 0.5%, 1%, and 2% (*w*/*w*). The extracts were mixed with the ground meat to ensure complete homogenisation. The poultry meat for each treatment was divided into 12 groups of 20 g, with three samples for each time of analysis. For the control group, three extra groups were prepared to analyse at day 0 to establish the initial physicochemical and microbiological conditions of the meat. All samples were placed in airtight, food-grade plastic containers and stored in a controlled refrigerator (5 ± 2 °C) for 15 days. The poultry meat was subjected to the following tests: microbial growth, lipid oxidation, pH, titratable acidity, moisture, total volatile basic nitrogen, and colour, carried out at the end of 0 (only control, without extract), 4, 8, 11, and 15 storage days. The assay was carried out in triplicate (technical replicates, derived from the same batch) for each storage time.

### 2.4. Physicochemical Characterization

#### 2.4.1. Moisture

The determination of moisture was conducted according to the AOAC [37]. In short, 1 g of sample was weighed in a previously weighed capsule, placed in an oven at 100 ± 2 °C, and weighed until the weight was constant.

#### 2.4.2. pH and Titratable Acidity

The pH and titratable acidity were measured following the AOAC [37]. First, 5 g of sample was mixed with 50 mL of water and homogenised for 15 min, and the pH of the solution was measured using a digital pH device (Crison micropH 2001). Then, the total titratable acidity was measured by titrating the same solution with 0.1 N sodium hydroxide (NaOH), using 0.1% phenolphthalein as an indicator. The outcome is presented as grams of oleic acid per 100 g of meat (g OA/100 g).

#### 2.4.3. Colour

The determination of the CIE-L* a* b* coordinates was used to assess the colour of the poultry meat samples. A CR 410 colorimeter (Minolta Co., Tokyo, Japan) with a D65 light source and a 10-degree visual angle were used for the measurement. The measurements were done on a standardized white background, and five shots were taken at different locations on each sample. The results are presented as the average of each of the coordinates (L*, a*, and b* values) and the colour difference (∆E) was determined following Formula (1), where L_C_*, a_C_*, and b_C_* are the colour parameters of the control sample on day 0 and L*, a*, and b* are the parameters of the samples on the following days.

(1) $$∆E=\sqrt{{\left(L_{C}^{*}-L^{*}\right)}^{2}+({a_{C}^{*}-a^{*})}^{2}+({b_{C}^{*}-b^{*})}^{2}}$$

#### 2.4.4. Total Volatile Basic Nitrogen

The total volatile basic nitrogen (TVB-N) was calculated according to Malle and Poumeyrol [38]. In summary, 25 g of sample was dissolved in 50 mL of 7.5% trichloroacetic acid before undergoing filtering through Whatman No.1 filter paper and then diluted. Phenolphthalein at 0.1% was used as an indicator, and to alkalize the mixture, sodium hydroxide (NaOH 6N) was put in. Next, 50 mL of 2% boric acid (20 g/L) and 0.5 mL of indicator solution were added to an Erlenmeyer flask, which collected the distillate, where the colour changed from purple to green. Ultimately, the distillate solution was titrated with hydrochloric acid (HCl 0.02 N) until the solution turned purple again. The result is presented as grams of nitrogen per 100 g of meat (g N/100 g).

### 2.5. Lipid Oxidation—Thiobarbituric Acid Reactive Substance (TBARS)

To evaluate the lipid oxidation of the sample, the thiobarbituric acid reactive substance method described by Souza et al. [39] was followed. To summarize, 15 g of sample was weighed, and 30 mL of trichloroacetic acid (TCA) 7.5% (*w*/*v*) was added. The mixture was stirred and then filtered with qualitative filter paper. Then, 5 mL of the solution was transferred to a test tube, and 5 mL of 0.02 M thiobarbituric acid (TBA) was applied. The tube was then placed in a water bath for 30 min at 90 °C, and, after cooling to room temperature, the absorbance was read at 530 nm in a UV/VIS spectrophotometer. The result is presented in mg of malondialdehyde (MDA) per kilogram of sample (mg MDA/kg) based on a calibration curve using 1,1,3,3-tetraethoxypropane, and the concentrations ranged from 0.0025 to 0.05 µmol.

### 2.6. Microbiological Growth

The microbial growth on the poultry meat was evaluated through the counting of total mesophilic aerobic microorganisms [40], total psychrotrophic aerobic microorganisms [41], and Enterobacteriaceae [42]. Briefly, 1 g of meat was mixed with 9 mL of saline solution (0.1% *m*/*v* of tryptone and 0.85% *m*/*v* of sodium chloride) and homogenised, and a series of decimal dilutions were carried out. Inoculation was performed in Petri dishes by adding 1 mL of the corresponding dilution and the culture medium. Plate count agar (PCA) medium was used for total mesophilic and total psychrotrophic aerobic microorganisms, and violet-red bile glucose (VRBG) medium was used for Enterobacteriaceae. After that, the Petri dishes were incubated in an oven at 30 °C for 72 h to count total viable mesophilic microorganisms and at 37 °C for 24 h for Enterobacteriaceae. Total viable psychrotrophic microorganisms were incubated at 7 °C for 168 h.

### 2.7. Statistical Analysis

The statistical analysis of the data was calculated with IBM^®^ SPSS^®^ Statistics, version 27.0.1.0 (Chicago, IL, USA), employing one-way analysis of variance (ANOVA) and ANOVA with repeated measures. The Tukey test was applied to examine the disparities among average values. The statistical significance level was determined at a value of *p* < 0.05, and the results are shown as the mean value accompanied by the standard deviation.

## 3. Results and Discussion

### 3.1. Physicochemical Characterization

#### 3.1.1. Moisture

The exchange of water between food and the environment is responsible for the preservation of certain foods. A high moisture content is significantly linked to multiple chemical, enzymatic, and microbiological deterioration processes [43,44]. Table 2 displays the results obtained for the moisture content of the studied samples. During the 15-day experiment, there was no significant impact on the moisture content of the poultry meat under the refrigeration conditions. This observation was consistent across both the control samples and the samples with extract. The control samples exhibited a marginal reduction in moisture content from 75.5% to 73.2% between the initial and final days of the experiment. In samples with extract, there was a slight increase in moisture content, with the exception of the poultry meat sample containing 2% (*w*/*w*) globe artichoke leaf extract, which exhibited a slight decrease from 74.2% to 73.2%. There were no statistically significant (*p* < 0.05) variations detected either within the samples or among the storage periods. Comparable results were reported by Brannan's [45] study, in which grape seed extract was applied to refrigerated ground chicken thigh meat. The study indicated that the moisture content remained consistent within the range of 70% to 80% [45].

#### 3.1.2. pH and Titratable Acidity

pH and acidity are indicators of food stability since they are associated with chemical reactions and microbiological growth, which lead to food deterioration. Food spoilage increases pH and reduces acidity values. The rise in pH during storage is due to the degradative mechanisms that lead to the formation of amines, ammonia, and organic sulphides. In meat, the pH increase changes the rate of biochemical reactions and creates favourable conditions for microbial growth. Furthermore, microbial proliferation results in the production of organic amines, also known as total volatile basic nitrogen (TVB-N), which raises the pH of the meat [28,46]. On average, the control samples had a higher pH than those with extracts (Table 2). The pH of the control sample increased throughout the storage days, despite slightly decreasing on the 11th day and significantly increasing (*p* < 0.05) again on the 15th day. Although the pH of the samples with extract increased slightly over the 15-day storage period, the changes were not statistically significant (*p* < 0.05). Comparing the control sample with the samples with extract, a statistically significant difference (*p* < 0.05) was observed after the 15-day period, where the control presented a pH of 7.62 and the samples with extract had an average value of 6.00 (5.99–6.15), with no statistically significant differences (*p* < 0.05) among them.

The maintenance of the pH level in the samples treated with the ethanolic extracts obtained from cultivated cardoon and globe artichoke leaves could potentially be correlated to the existence of phenolic compounds within their composition, such as caffeoylquinic acids and apigenin and luteolin derivatives [39,46,47,48]. The presence of these compounds in these species’ leaves has been identified as the main reason for the antimicrobial activity of the extracts. Consequently, through these compounds, the extract effectively inhibits microbial growth, reduces the release of volatile compounds, and prevents lipid oxidation in the samples, thereby maintaining stable pH levels [39,46,47,48]. At the conclusion of the storage period, it was observed that the control sample exhibited a reduced level of acidity (2.85 g OA/100 g) in contrast to the samples with the extracts (3.40–4.00 g OA/100 g), which was in accordance with the pH results (Table 2). The acidity of the control sample initially had an increasing trend, but from day 11, it began to decrease. After 15 days, the acidity of samples containing extract remained essentially stable, with no significant differences. The findings presented in the present study are in accordance with those stated by Paglarini et al. [28] (Table 1), who carried out an investigation on the antioxidant properties of mechanically deboned poultry meat using five distinct extracts (namely, grape, rosemary, green tea, mate, and pomegranate extract) at different concentrations. The results of this investigation indicate that the inclusion of extracts in poultry meat led to a reduction in pH levels when compared to the control group. However, the pH values present in this study were lower than those obtained in the present study. Bigolin et al. [49] evaluated the influence of ascorbic acid, sodium chloride, and sodium erythorbate on poultry meat. Despite verifying a slight increase in pH, the values are the same as those obtained in this study. Furthermore, their observations noted that the added antioxidant compounds effectively prevented an increase in alkalinity, a phenomenon aligned with the findings of this study [49].

#### 3.1.3. Colour

Meat colour is a significant factor in assessing its quality; it is supposed to remain unchanged during the storage time. Colour is an important factor, as it strongly influences the consumer’s choice [50]. All extract concentrations affected the poultry meat’s colour after their addition (Table 3). Higher ∆E values (*p* < 0.05) were obtained for all concentrations during the course of the experiment, as compared to the control. Likewise, the higher the extract concentration, the greater the colour difference. The colour difference can be explained by the content of polyphenolic compounds, namely hydroxycinnamic acids and flavonoids, in the extracts. These compounds are responsible for several properties of the extracts, including their colour. Moreover, cardoon has in its constitution anthocyanin pigments that are also responsible for the colour of the leaves. Usually, cultivated cardoon leaves present a grey-yellow colour and globe artichoke leaves a deep green colour. However, the colour of the leaves depends on environmental factors, namely the sunlight to which the plant was exposed [47,51,52,53,54]. All concentrations of cultivated cardoon extract presented lower (*p* < 0.05) brightness (L* values) (Table 3) in comparison to the control during the storage period. Globe artichoke at 0.5% and 1% presented higher (*p* < 0.05) brightness in contrast to the control, whereas 2% globe artichoke presented lower values. Regarding the a* values (red/green colour), the control presented statistically significant (*p* < 0.05) higher a* values as compared to the samples with extract, indicating a greener colour attributed to the leaf extract (Table 3). Nonetheless, for the samples with cultivated cardoon extracts, the a* values increased during the storage period, whereas for the globe artichoke, they decreased. As regards the b* values (blue/yellow colour), they remained practically constant within the samples, with significant differences (*p* < 0.05) just for 1% and 2% cultivated cardoon extracts (Table 3), probably due to the yellowish colour of the cultivated cardoon leaves. Samples with cultivated cardoon extracts presented higher b* values, indicating a more yellow colour, while the control samples presented lower b* values during storage time. Other studies also obtained similar results, in which different extracts that were applied influenced the colour of different meat products [28,55,56]. For example, in the Paglarini et al. [28] (Table 1) study, the extract affected the colour of the poultry meat. The green tea extract added to the poultry meat lowered a* values, giving the meat a greener colour. The b* value also decreased, giving a more yellowish colour, possibly because of the yellow-green colour of the extract [28]. Basanta et al. [13] (Table 1) concluded that the extracts helped to preserve the colour of the chicken patties during the storage time. All a* values were above zero, indicating the red colour of the patties, and they were significantly higher for the patties with peel extract, compared to the control [13].

#### 3.1.4. Total Volatile Basic Nitrogen

Spoiling mechanisms lead to the deterioration of proteins, and other nitrogen-containing substances lead to the formation of organic amines, also identified as total volatile basic nitrogen (TVB-N). These chemicals pose a risk to food safety, and they are responsible for significant colour and flavour modifications in meat and its derivatives. So, it is expected that the TVB-N of poultry meat will increase during storage time due to natural spoilage mechanisms [57]. The TVB-N significantly increased (*p* < 0.05) for the control group during storage, as shown in Table 2. The TVB-N content was 23 mg N/100 g meat at the beginning, indicating it was fresh, and by the 15th day, it had reached 278 mg N/100 g meat, suggesting a significant deterioration in quality. Nonetheless, the TVB-N value of the samples with extracts remained stable throughout storage (Table 2), showing no significant increase over time within each treatment (*p* < 0.05). At all storage time points, the TVB-N values in the treated samples were significantly lower than those of the control group (*p* < 0.05), indicating the effectiveness of the extracts in slowing protein degradation.

During the natural process of degradation of the meat, the microorganisms degrade the proteins, and consequently, there is a release of nitrogen in the environment, thus increasing the TVB-N value. However, this was not observed in the samples with extracts. These results can be explained by the presence of caffeoylquinic acids in cultivated cardoon and globe artichoke leaves. These compounds exhibit antimicrobial activity, and consequently, there is less microbiological degradation of the meat, less degradation of the protein, and subsequently less release of volatile nitrogen [47]. Bekhit et al. [57] state that the freshness of poultry meat is determined by a TVB-N value between 25 and 29 mg N/100 g meat. Therefore, it seems that the extracts were efficient in delaying the meat’s degradation and preserving its freshness. These results are corroborated by the ones obtained by Saleh et al. [25] (Table 1). The researchers evaluated the effect of olive leaf extract at different concentrations on poultry meat slices, and concluded that the extract was effective at reducing the TVB-N values, specifically at the concentration of 1% [25].

### 3.2. Lipid Oxidation—Thiobarbituric Acid Reactive Substance (TBARS)

Lipid oxidation is among the primary sources of food spoilage. It is a natural occurrence responsible for food's off-flavours, loss of nutritional value, and texture and colour changes, leading to its decreased shelf life and consumer rejection. High levels of PUFA, particularly prone to lipid oxidation, are a distinctive feature of poultry meat [58]; thus, it was chosen as the food matrix to assess the effectiveness of cultivated cardoon and globe artichoke leaf extracts in retarding its lipid oxidation. By the end of the 15-day storage period, it was observed that the control sample obtained significantly higher (*p* < 0.05) MDA values than the samples with extracts in all concentrations (Table 2). The MDA values for the control samples exhibited a significant increase (*p* < 0.05) throughout the storage duration, rising from 0.256 mg MDA/kg meat to 0.745 mg MDA/kg meat. However, there was only a slight increase for the samples with extracts, lacking statistical significance (*p* < 0.05). Unlike the control group, which reached 0.5 mg MDA/kg meat on the 11th day of storage, all MDA values for the samples with extracts were less than 0.5 mg MDA/kg meat, the minimum acceptable amount to detect a sense of off-flavour [59]. In this sense, cultivated cardoon and globe artichoke leaf extracts were successful in delaying poultry meat’s lipid oxidation. This delay is due to the presence of bioactive compounds, namely flavonoids (apigenin and luteolin derivatives) and caffeoylquinic acids (chlorogenic acid), which confer the antioxidant activity presented by these extracts, limiting the formation of MDA [4,47]. No significant differences (*p* < 0.05) were observed among the different concentrations of extracts over the 15 days of storage, although the cultivated cardoon leaf extract showed greater antioxidant capacity than the globe artichoke leaf extract [23]. Nonetheless, poultry meat mixed with 1% cultivated cardoon leaf extract presented the lowest MDA value after a 15-day storage period. Table 1 presents several studies where natural extracts successfully protected poultry meat against lipid oxidation, tested through thiobarbituric acid reactive substance (TBARS) assays. For instance, Paglarini et al. [28] obtained comparable results, where the lipid oxidation was delayed in the samples treated with plant extracts as compared to the control sample. The MDA values were significantly higher in the control sample, indicating the efficacy of plant extracts in preventing lipid oxidation. The findings of Saleh et al. [25] and Ahmad et al. [30] also align with these results, as their plant extracts helped to maintain the TBA values low throughout the storage period.

### 3.3. Microbiological Growth

Table 4 summarizes the microbial growth observed during the storage time for *Enterobacteriaceae*, total mesophilic aerobic microorganisms, and total psychrotrophic aerobic microorganisms. The control sample presented microbial growth, which was expected given poultry’s natural degradation. Overall, the control sample presented a significantly higher (*p* < 0.05) total count for all microorganisms than the treated samples on all days.

The total mesophilic aerobic microorganisms’ initial count was 4.86 log CFU/g meat, reaching its maximum (13.33 log CFU/g meat) on the 11th day of storage. By the end of the experiment (day 15), it was observed that the count was decreasing, indicating that the microorganisms had nothing left to degrade and began to die. Regarding the samples with extract, all reached their maximum count on the 8th day of storage. Nonetheless, counts on the 8th day were close to the initial counts, showing that the extracts efficiently inhibited microbial growth. On days 11 and 15, the counts decreased as they did for the control samples. Yet, among the samples with extracts, the 0.5% cultivated cardoon sample presented the highest count (5.05 log CFU/g meat) of total aerobic microorganisms. Mazzaglia et al. [19], who produced aubergine burgers with 1% and 3% of *Cynara cardunculus* extracts, achieved identical results, as they showed a growth suppression of total mesophylls at both concentrations of extract in comparison to the control. The total psychrotrophic aerobic microorganisms’ count exhibited a similar development rate to that of the total mesophilic aerobic microorganisms. The control sample started with a count of 3.43 log CFU/g meat and peaked at 10.54 log CFU/g meat after the conclusion of day 11 of storage. Additionally, it presented a significantly higher total count (*p* < 0.05) in contrast to the samples with extract. Regarding the samples with extract, there were no statistically significant differences (*p* < 0.05) in the counts over storage. Nevertheless, the counts of samples with extracts remained almost identical to the initial counts of the samples, demonstrating that the extract was successful at preventing the growth of total psychrotrophs at all concentrations. Mazzaglia et al. [19] also tested the aubergine burgers for total psychrotrophic microorganisms and concluded that the extract could inhibit the growth of psychrophilic bacteria. Therefore, their study is in line with ours since the extracts inhibited the growth of total psychrotrophs in poultry meat.

Finally, regarding the *Enterobacteriaceae* count, as expected, the control samples presented a significantly higher (*p* < 0.05) count in contrast to the samples with extracts throughout storage time. The count of *Enterobacteriaceae* for the control samples went from 2.36 log CFU/g meat on day 1 to 11.18 log CFU/g meat (maximum count) on day 11. In samples with the extract, the growth of *Enterobacteriaceae* was slow, indicating that the extract can inhibit the development of this microorganism.

**Figure 1:**
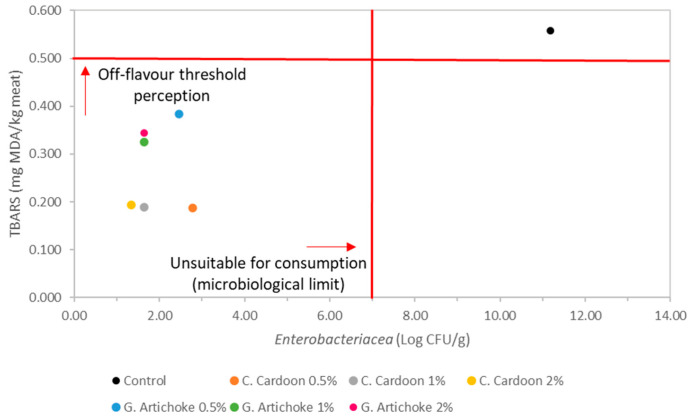
Plot of the microbiology study (*Enterobacteriaceae*) and the lipid oxidation results of poultry meat mixed with different concentrations of cultivated cardoon leaf extract and globe artichoke leaf extract on day 11 of the storage time.

To determine the best concentration of extract to be applied to poultry meat, the growth of *Enterobacteriaceae* was related to the MDA values on day 11 (Figure 1). By analysing Figure 1, it can be seen that there were not many differences between the different concentrations of the same extract. However, the cultivated cardoon extract showed better results than the globe artichoke extract, despite both remaining within acceptable limits. The cultivated cardoon extract at concentrations of 1% (*w*/*w*) and 2% (*w*/*w*) showed the best results, but when compared to the remaining results, the cultivated cardoon extract at 1% (*w*/*w*) presents as the best option since it does not have such a pronounced yellowish-green colour (Table 2). The microbiological outcomes corroborate the physicochemical results, since the extracts were able to maintain constant pH and acidity levels, delaying the microbial growth and minimising the release of TVB-N. These results can be supported by the antimicrobial and antioxidant activity that these extracts have due to their composition of polyphenolic compounds, namely caffeoylquinic acids, apigenin, luteolin, and chlorogenic acid, as previously mentioned [4,47]. Similar results, where different plant extracts delayed microbial growth when mixed with poultry meat, were also obtained by several authors (Table 1). For example, Dubeni et al. [27] evaluated the potential of *Moringa oleifera* leaf extracts to inhibit microbial growth in chicken breasts. The authors studied the effects of *Moringa oleifera* leaf extracts at different concentrations (0.25%, 0.5%, 0.7%, and 1%), and concentrations of 0.25% and 0. 50% were the most effective in controlling microbial growth. The extract was effective against aerobic count, *E. coli*, *L. monocytogenes*, and *Salmonella* spp.

Cardoon leaf extracts demonstrated promising antioxidant and antimicrobial properties for poultry meat preservation, but their application in the food industry remains limited due to several critical factors. These include challenges related to maintaining meat colour, which may affect acceptability by consumers, determining the optimal dosage, and obtaining comprehensive toxicological validation. Furthermore, under current EU regulations, the use of such extracts as food additives is not yet authorized. According to EFSA guidelines, introducing novel ingredients into food products requires extensive toxicological data submission, encompassing assessments of genotoxicity, subchronic toxicity, and metabolite profiling. Therefore, future research must address not only the efficacy but also the safety, standardization, and regulatory compliance aspects to facilitate the potential commercialization of cardoon leaf extracts in meat products.

## 4. Conclusions

Different concentrations of extracts were applied, and all were efficient at preserving the quality and stability of the poultry meat during the 15-day refrigerated period in comparison with the control. The results suggest that the extracts maintained a stable pH and acidity level, which inhibited microbial development. The addition of *C*. *cardunculus* to poultry meat significantly reduced microbial growth compared to control samples, limiting the emission of volatile basic nitrogen by the conclusion of the experiment (day 15). In addition, the extracts successfully reduced the lipid oxidation of poultry meat in contrast to the control samples over the storage time. Due to the green colour of the extract that is transferred to the poultry meat, the colour of the extract can be a restrictive feature for consumer acceptance of the product due to its colouration. This effect was confirmed by the ΔE values, particularly at 1% and 2% concentrations of both extracts, indicating that the colour change would likely be perceptible to the naked eye. Also, the increase in concentration of the extract resulted in a greater colour intensity of the extract being transferred to the poultry meat. Overall, poultry meat with 1% (*w*/*w*) of cultivated cardoon leaf extract presented the most promising results. It is necessary to assess the toxicity of these natural extracts to determine whether they may be used as food additives, and it is also important to define the bioactive(s) compound(s) that should be used to standardize the cardoon extracts and their amounts, as a way to ensure the quality and effectiveness of these extracts. Further research should apply cardoon extracts to other food matrices (e.g., fish products) in order to evaluate their ability to extend food shelf life.

## Figures and Tables

**Table 2:** Physicochemical characterization of the poultry meat with different extracts during 15 days of storage.

Assays	Days	Control	Cultivated Cardoon			Globe Artichoke		
			0.5%	1%	2%	0.5%	1%	2%
Moisture (%)	0	75.5 ± 0.0 ^A^	-	-	-	-	-	-
	4	75.0 ± 0.6 ^aA^	76.5 ± 1.1 ^aA^	75.4 ± 0.0 ^aA^	74.7 ± 0.2 ^aA^	75.8 ± 0.5 ^aA^	75.5 ± 0.5 ^aA^	74.2 ± 0.9 ^aA^
	8	75.7 ± 0.2 ^aA^	76.3 ± 0.2 ^aA^	76.4 ± 0.2 ^aA^	75.5 ± 0.6 ^aA^	76.9 ± 0.7 ^aA^	75.8 ± 0.3 ^aA^	75.5 ± 0.1 ^aA^
	11	76.9 ± 0.7 ^aA^	76.9 ± 0.7 ^aA^	76.4 ± 0.7 ^aA^	76.0 ± 0.4 ^aA^	76.2 ± 0.7 ^aA^	76.1 ± 0.9 ^aA^	75.3 ± 0.6 ^aA^
	15	73.2 ± 5.3 ^aA^	77.0 ±1.5 ^aA^	76.8 ± 1.3 ^aA^	76.0 ± 0.4 ^aA^	77.0 ± 0.2 ^aA^	76.6 ± 0.9 ^aA^	73.7 ± 2.8 ^aA^
pH	0	5.96 ± 0.01 ^B^	-	-	-	-	-	-
	4	6.07 ± 0.04 ^abB^	6.00 ± 0.04 ^abA^	6.08 ± 0.00 ^abA^	6.18 ± 0.13 ^bA^	5.96 ± 0.01 ^aA^	6.00 ± 0.04 ^abA^	5.95 ± 0.00 ^aA^
	8	6.16 ± 0.00 ^aB^	6.10 ± 0.04 ^abA^	6.10 ± 0.01 ^abA^	6.04 ± 0.01 ^bcA^	6.02 ± 0.03 ^cA^	5.98 ± 0.00 ^cdA^	5.93 ± 0.01 ^dA^
	11	5.95 ± 0.21 ^aB^	6.03 ± 0.04 ^aA^	6.10 ± 0.06 ^aA^	6.06 ± 0.01 ^aA^	6.02 ± 0.06 ^aA^	6.07 ± 0.08 ^aA^	6.02 ± 0.13 ^aA^
	15	7.62 ± 0.21 ^aA^	6.06 ± 0.01 ^bA^	6.15 ± 0.12 ^bA^	6.01 ± 0.01 ^bA^	6.02 ± 0.01 ^bA^	6.06 ± 0.08 ^bA^	5.99 ± 0.06 ^bA^
Titratable acidity (g of oleic acid/100 g of meat)	0	3.80 ± 0.00 ^AB^	-	-	-	-	-	-
	4	4.05 ± 0.07 ^aA^	4.00 ± 0.14 ^aA^	3.80 ± 0.00 ^aA^	3.80 ± 0.28 ^aA^	4.20 ± 0.14 ^aA^	4.30 ± 0.14 ^aA^	4.05 ± 0.21 ^aA^
	8	4.25 ± 0.07 ^abcA^	4.10 ± 0.00 ^aA^	4.15 ± 0.07 ^abA^	4.50 ± 0.14 ^cA^	4.25 ± 0.07 ^abcA^	4.25 ± 0.07 ^abcA^	4.40 ± 0.00 ^bcA^
	11	3.85 ± 0.64 ^aAB^	3.80 ± 0.42 ^aA^	3.80 ± 0.57 ^aA^	4.05 ± 0.92 ^aA^	3.95 ± 0.35 ^aA^	3.95 ± 0.49 ^aA^	3.95 ± 0.78 ^aA^
	15	2.85 ± 0.07 ^aB^	3.50 ± 0.28 ^aA^	3.40 ± 1.14 ^aA^	3.90 ± 0.57 ^aA^	3.95 ± 0.21 ^aA^	3.90 ± 0.14 ^aA^	4.00 ± 0.28 ^aA^
TVB-N (mg N/100 g meat)	0	23 ± 1 ^D^	-	-	-	-	-	-
	4	125 ± 5 ^aB^	22 ± 1 ^bA^	24 ± 1 ^bA^	23 ± 0 ^bA^	23 ± 2 ^bA^	24 ± 1 ^bA^	25 ± 1 ^bA^
	8	72 ± 0 ^aC^	24 ± 4 ^bA^	24 ± 1 ^bA^	24 ± 1 ^bA^	24 ± 1 ^bA^	24 ± 1 ^bA^	26 ± 0 ^bA^
	11	101 ± 10 ^aB^	21 ± 1 ^bA^	23 ± 2 ^bA^	25 ± 2 ^bA^	21 ± 4 ^bA^	22 ± 1 ^bA^	24 ± 1 ^bA^
	15	278 ± 10 ^aA^	23 ± 2 ^bA^	24 ± 0 ^bA^	26 ± 3 ^bA^	24 ± 1 ^bA^	21 ± 0 ^bA^	24 ± 1 ^bA^
TBARS (mg MDA/Kg meat)	0	0.26 ± 0.14 ^A^	-	-	-	-	-	-
	4	0.21 ± 0.05 ^aA^	0.28 ± 0.00 ^aA^	0.22 ± 0.01 ^aA^	0.24 ± 0.01 ^aA^	0.26 ± 0.01 ^aA^	0.30 ± 0.16 ^aA^	0.32 ± 0.08 ^aA^
	8	0.27 ± 0.12 ^aA^	0.24 ± 0.00 ^aA^	0.26 ± 0.06 ^aA^	0.24 ± 0.01 ^aA^	0.25 ± 0.06 ^aA^	0.33 ± 0.03 ^aA^	0.36 ± 0.00 ^aA^
	11	0.56 ± 0.23 ^aA^	0.19 ± 0.05 ^aA^	0.19 ± 0.06 ^aA^	0.19 ± 0.03 ^aA^	0.38 ± 0.08 ^aA^	0.33 ± 0.05 ^aA^	0.35 ± 0.03 ^aA^
	15	0.75 ± 0.13 ^aA^	0.35 ± 0.07 ^bA^	0.22 ± 0.06 ^bA^	0.25 ± 0.01 ^bA^	0.38 ± 0.03 ^bA^	0.39 ± 0.08 ^bA^	0.41 ± 0.06 ^bA^

TVB-N = total volatile basic nitrogen. TBARS = thiobarbituric acid reactive substance. The lowercase letters compare samples within the same storage day. The uppercase letters compare samples with the same amount of extract for different storage days. Different letters, for either lowercase or uppercase comparison, represent results with significant differences (*p* < 0.05) by the Tukey test.

**Table 3:** Colour values of the poultry meat samples with different extracts during 15 days of storage.

CIE L*, a*, b* Values	Days	Control	Cultivated Cardoon			Globe Artichoke		
			0.5%	1%	2%	0.5%	1%	2%
L*	0	61.35 ± 0.07 ^A^	-	-	-	-	-	-
	4	61.53 ± 1.70 ^aA^	58.51 ± 1.40 ^abA^	52.58 ± 0.96 ^cdB^	49.97 ± 0.78 ^dB^	62.64 ± 0.41 ^aB^	59.25 ± 0.83 ^abA^	56.23 ± 1.09 ^bcA^
	8	61.02 ± 1.48 ^aA^	60.29 ± 0.76 ^abA^	54.44 ± 0.78 ^cdAB^	51.89 ± 1.07 ^dAB^	64.09 ± 0.58 ^aAB^	60.56 ± 1.06 ^abA^	57.91 ± 0.84 ^bcA^
	11	61.73 ± 6.01 ^abA^	60.83 ± 0.04 ^abA^	55.72 ± 0.28 ^abA^	53.20 ± 0.18 ^bA^	64.25 ± 0.79 ^aAB^	60.86 ± 0.49 ^abA^	57.58 ± 1.37 ^abA^
	15	60.99 ± 1.36 ^aA^	58.88 ± 3.68 ^bcA^	56.47 ± 0.77 ^bcA^	53.09 ± 0.57 ^cA^	65.72 ± 0.62 ^aA^	61.66 ± 0.40 ^abA^	58.29 ± 0.59 ^bcA^
a*	0	9.65 ± 0.76 ^A^	-	-	-	-	-	-
	4	8.69 ± 0.34 ^aA^	−2.97 ± 0.11 ^eB^	−6.77 ± 0.01 ^fA^	−8.48 ± 0.13 ^gA^	3.07 ± 0.23 ^bA^	1.49 ± 0.66 ^cA^	−0.65 ± 0.06 ^dA^
	8	9.36 ± 0.79 ^aA^	−2.57 ± 0.32 ^dAB^	−6.50 ± 0.11 ^eA^	−7.86 ± 0.16 ^eA^	2.07 ± 0.08 ^bB^	0.52 ± 0.42 ^cA^	−0.81 ± 0.01 ^cA^
	11	7.55 ± 1.53 ^aA^	−1.92 ± 0.06 ^cAB^	−5.51 ± 1.06 ^dA^	−6.85 ± 0.95 ^dA^	2.07 ± 0.22 ^bB^	0.69 ± 0.60 ^bcA^	−0.89 ± 0.19 ^bcA^
	15	10.14 ± 1.70 ^aA^	−1.70 ± 0.52 ^cA^	−4.96 ± 0.57 ^dA^	−6.48 ± 0.33 ^dA^	1.47 ± 0.35 ^bB^	0.35 ± 0.31 ^bcA^	−0.89 ± 0.37 ^bcA^
b*	0	13.87 ± 0.21 ^A^	-	-	-	-	-	-
	4	13.48 ± 0.93 ^cA^	17.81 ± 0.24 ^aA^	17.99 ± 0.04 ^aC^	18.17 ± 0.14 ^aB^	14.79 ± 0.21 ^bcA^	15.04 ± 0.01 ^bA^	15.99 ± 0.01 ^bA^
	8	13.05 ± 0.85 ^cA^	17.86 ± 0.11 ^aA^	18.48 ± 0.08 ^aB^	18.72 ± 0.04 ^aAB^	14.72 ± 0.38 ^bA^	15.16 ± 0.11 ^bA^	15.90 ± 0.26 ^bA^
	11	14.00 ± 1.71 ^cA^	17.59 ± 0.21 ^abA^	18.76 ± 0.03 ^aC^	18.96 ± 0.31 ^aA^	14.98 ± 0.74 ^bcA^	15.25 ± 0.06 ^bcA^	15.81 ± 0.31 ^bcA^
	15	14.01 ± 0.46 ^cA^	17.88 ± 0.23 ^aA^	18.58 ± 0.03 ^aBC^	19.22 ± 0.14 ^aA^	14.90 ± 0.69 ^bcA^	15.24 ± 0.25 ^bcA^	16.19 ± 0.60 ^bA^
∆E	4	1.95 ± 0.47 ^eB^	13.54 ± 1.02 ^bA^	19.07 ± 1.11 ^aA^	21.83 ± 0.91 ^aA^	6.78 ± 0.52 ^dA^	8.52 ± 0.31 ^cdA^	11.70 ± 1.09 ^bcA^
	8	1.93 ± 0.49 ^eB^	12.90 ± 0.50 ^bA^	18.16 ± 0.88 ^aA^	20.48 ± 1.03 ^aA^	8.13 ± 0.67 ^dA^	9.28 ± 0.43 ^cdA^	11.20 ± 1.02 ^bcA^
	11	5.15 ± 1.44 ^dA^	12.16 ± 0.79 ^bA^	16.90 ± 0.16 ^aA^	19.09 ± 0.15 ^aA^	8.22 ± 1.34 ^cdA^	9.09 ± 0.22 ^bcA^	11.38 ± 1.05 ^bcA^
	15	2.09 ± 0.79 ^eAB^	12.57 ± 0.38 ^cA^	16.12 ± 0.45 ^bA^	18.90 ± 0.69 ^aA^	9.36 ± 0.21 ^dA^	9.41 ± 0.50 ^dA^	11.22 ± 0.66 ^cdA^

L* = brightness. a* = red/green colour. b* = blue/yellow colour. ∆E = colour difference. The lowercase letters compare samples within the same storage day. The uppercase letters compare samples with the same amount of extract for different storage days. Different letters, for either lowercase or uppercase comparison, represent results with significant differences (*p* < 0.05) by the Tukey test.

**Table 4:** Microbiological growth of poultry meat during storage time.

Assays	Days	Control	Cultivated Cardoon			Globe Artichoke		
			0.5%	1%	2%	0.5%	1%	2%
Total mesophilic aerobic microorganisms (Log CFU/g meat)	0	4.86 ± 0.06 ^D^	-	-	-	-	-	-
	4	7.69 ± 0.08 ^aC^	3.97 ± 0.71 ^bA^	4.08 ± 0.52 ^bA^	4.13 ± 0.40 ^bA^	3.94 ± 0.51 ^bA^	3.55 ± 0.89 ^bA^	3.99 ± 0.74 ^bA^
	8	10.55 ± 0.78 ^aB^	5.05 ± 1.31 ^bA^	4.51 ± 0.71 ^bA^	4.45 ± 0.50 ^bA^	4.43 ± 0.77 ^bA^	4.65 ± 0.52 ^bA^	4.34 ± 0.61 ^bA^
	11	13.33 ± 0.57 ^aA^	3.95 ± 0.34 ^bA^	3.62 ± 0.37 ^bA^	3.30 ± 1.00 ^bA^	3.47 ± 1.00 ^bA^	2.83 ± 0.83 ^bA^	3.30 ± 1.30 ^bA^
	15	10.98 ± 0.00 ^aAB^	3.38 ± 0.43 ^bA^	2.72 ± 0.24 ^bA^	3.30 ± 0.61 ^bA^	2.93 ± 0.63 ^bA^	3.06 ± 0.37 ^bA^	4.13 ± 0.00 ^bA^
Total psychrotrophic aerobic microorganisms (Log CFU/g meat)	0	3.43 ± 0.20 ^B^	-	-	-	-	-	-
	4	7.49 ± 0.34 ^aA^	3.75 ± 0.20 ^bA^	3.94 ± 0.29 ^bA^	3.92 ± 0.19 ^bA^	3.43 ± 0.77 ^bA^	3.40 ± 0.74 ^bA^	3.86 ± 0.43 ^bA^
	8	10.48 ± 0.92 ^aA^	4.89 ± 1.04 ^bA^	4.06 ± 0.63 ^bA^	3.32 ± 0.67 ^bA^	3.46 ± 0.20 ^bA^	3.54 ± 0.11 ^bA^	3.31 ± 0.35 ^bA^
	11	10.54 ± 0.90 ^aA^	4.00 ± 0.30 ^bA^	4.04 ± 0.56 ^bA^	4.22 ± 0.56 ^bA^	4.70 ± 0.22 ^bA^	3.74 ± 0.09 ^bA^	4.05 ± 0.25 ^bA^
	15	10.54 ± 0.90 ^aA^	3.96 ± 0.11 ^bA^	3.59 ± 0.15 ^bA^	3.85 ± 0.10 ^bA^	3.89 ± 0.23 ^bA^	3.75 ± 0.05 ^bA^	3.99 ± 0.34 ^bA^
*Enterobacteriaceae* (Log CFU/g meat)	0	2.36 ± 0.17 ^D^	-	-	-	-	-	-
	4	5.81 ± 0.00 ^aC^	1.64 ± 0.21 ^bA^	1.19 ± 0.24 ^bA^	0.95 ± 0.00 ^bA^	2.19 ± 0.54 ^bA^	2.06 ± 0.33 ^bA^	1.61 ± 0.65 ^bA^
	8	9.59 ± 0.00 ^aB^	3.36 ± 1.81 ^bA^	1.19 ± 0.24 ^bA^	0.95 ± 0.00 ^bA^	2.32 ± 0.32 ^bA^	1.93 ± 0.28 ^bA^	1.43 ± 0.00 ^bA^
	11	11.18 ± 0.52 ^aA^	2.78 ± 0.65 ^bA^	1.64 ± 0.39 ^bA^	1.34 ± 0.39 ^bA^	2.47 ± 0.19 ^bA^	1.64 ± 0.09 ^bA^	1.64 ± 0.21 ^bA^
	15	8.77 ± 0.00 ^aB^	3.19 ± 0.37 ^bA^	2.22 ± 0.04 ^bA^	2.33 ± 0.47 ^bA^	2.75 ± 0.29 ^bA^	1.83 ± 0.03 ^bA^	2.09 ± 0.65 ^bA^

The lowercase letters compare samples within the same storage day. The uppercase letters compare samples with the same amount of extract for different storage days. Different letters, for either lowercase or uppercase comparison, represent results with significant differences (*p* < 0.05) by the Tukey test.

## Data Availability

The original contributions presented in this study are included in the article. Further inquiries can be directed to the corresponding authors.
